# In Vivo Anti-Inflammatory Evaluation and In Silico Physicochemical Characterization of Flavanones from *E. platycarpa* Leaves

**DOI:** 10.3390/molecules30183728

**Published:** 2025-09-13

**Authors:** Berenice Andrade-Carrera, Valeri Domínguez-Villegas, Ana Cristina Calpena, María Luisa Garduño-Ramírez

**Affiliations:** 1 Facultad de Nutrición, Universidad Autónoma del Estado de Morelos, Cuernavaca 62350, Morelos, Mexico; 2 Facultad de Ciencias Químicas e Ingeniería, Universidad Autónoma del Estado de Morelos, Cuernavaca 62210, Morelos, Mexico; 3 Department of Pharmacy, Pharmaceutical Technology and Physical Chemistry, School of Pharmacy and Food Sciences, University of Barcelona, 27-31 Joan XXIII Av., 08028 Barcelona, Spain; 4 Centro de Investigaciones Químicas, Instituto de Investigación en Ciencias Básicas y Aplicadas, Universidad Autónoma del Estado de Morelos, Cuernavaca 62210, Morelos, Mexico; lgarduno@uaem.mx

**Keywords:** flavanones, *Eysenhardtia platycarpa*, anti-inflammatory activity, SAR

## Abstract

The inflammatory response is a defence mechanism triggered by tissue damage, aiming to eliminate harmful agents and initiate healing. Conventional anti-inflammatory drugs, such as NSAIDs and corticosteroids, are widely used but often cause severe side effects. Flavonoids, particularly flavanones, have shown significant anti-inflammatory activity with fewer adverse effects. In this study, eight analogues (**1a**–**1d**) and (**2a**–**2d**) were obtained from natural flavanones (**1**) and (**2**) using a pharmacomodulation strategy. NMR, FTIR, structurally confirmed all compounds and MS. Theoretical physicochemical analyses, including molecular orbital energies, dipole moments, and Log P, suggested favourable drug-like properties for these analogues. The anti-inflammatory activity was evaluated in vivo using a TPA-induced mouse ear edema model. Analogue (**2c**) exhibited the highest inhibition (98.62 ± 1.92%), followed by (**2d**) (76.12 ± 1.74%) and (**1c**) (71.64 ± 1.86%). Notably, structural modifications such as cyclization, methoxylation, and prenylation were associated with increased lipophilicity and biological activity, suggesting that tuning physicochemical properties may enhance pharmacological efficacy while preserving drug-likeness. Overall, these findings highlight semi-synthetic derivatization of flavanones as a valuable approach for developing potent and selective anti-inflammatory agents, positioning analogue (**2c**) as a promising lead for further pharmacological development.

## 1. Introduction

Inflammation represents the immune system’s response to protect the organism against foreign harmful stimuli including pathogens, damaged cells, toxic compounds and irradiation. Inflammation is primarily classified as acute and chronic [1]. One of the very firsts to define the parameters of inflammation was Aulus Conrenius Celsus (25 BD-50 AC), a Roman encyclopedist to whom we owe the famous statement, “*Notae vero inflammationis sunt quatuor: rubor et tumor cum calore and dolore*” (The signs of inflammation are four: redness, swelling, fever and pain) [2].

The inflammatory process produces a series of chemical mediators are activated that cause an increase in vascular permeability, leukocyte infiltration, followed by the synthesis of arachidonic acid metabolites, release of cytokines and adhesion molecules that cause the stimulation of other mediators and start chemotaxis [3]. If the tissue that undergoes an inflammatory process does not reestablish itself, the inflammatory cascade initiates a potential destruction of the damaged tissue that can last for months or years and. If not adequately controlled, can cause numerous diseases including rheumatoid arthritis, cancer and multiple sclerosis, among others [1].

Current therapy for inflammatory diseases is limited to the steroidal and non-steroidal anti-inflammatory agents. However, prolonged use of these drugs may produce many adverse effects. There is an important need to develop new anti-inflammatory agents with selective action and fewer side effects.

Flavonoids are plant secondary metabolites that are ubiquitous in fruits, vegetables, seeds, and plants. These polyphenolic compounds possess a basic 15-carbon skeleton and can be represented as C_6_-C_3_-C_6_ [4]. Flavonoids are known to exhibit several properties, including anti-inflammatory, antimutagenic, antioxidant, and anticancer effects [5].

The structural substitution in the flavonoid ring system is relevant to test their biological properties in search of more potent drug candidates. The chemical nature of flavonoids depends on their structural class, degree of hydroxylation, other substitutions and conjugations, and degree of polymerization [6].

Although there is a considerable amount of data in the literature, it remains challenging to identify a clear trend that would lead to an optimized structure with high activity and low toxicity [7]. Prenylation, for example, increases the lipophilicity of flavonoids, which enhances their affinity for biological membranes and improves their interaction with target proteins [1].

To further enhance the pharmacological efficacy of natural prenylated flavanones, different structural modifications are being explored as a strategy to develop analogues that are more effective. For instance, Andrade-Carrera, B. et al. synthesized a series of analogues from the flavanone (2*S*)-5,7-Dihydroxy-6-prenyl-flavanone, a compound isolated from methanolic extract of leaves of *Eysenhardtia platycarpa* using the pharmacomodulation approach. These compounds exhibited cytotoxic activity and were evaluated both in their free form and encapsulated in polymeric nanoparticles [8].

Regulatory mechanisms that contribute the progression of inflammation to cancer, it plays a crucial role in altering the tumour microenvironment, which in turn promotes tumour proliferation and rapid progression [9]. Consequently, many studies have suggested that preventing or inhibiting chronic inflammatory processes could be a key strategy for reducing the risk of chronic diseases, including cancer [10].

In this context, Dominguez-Villegas, V. et al. reported the in vivo anti-inflammatory activity of prenylated flavanones, isolated from the methanolic extract of leaves of *Eysenhardtia platycarpa*, using an inflammatory induction model in mouse ear tissue [11].

Building on these findings, there is growing interest in investigating the critical structural features of prenylated flavanones for the design of new, more potent anti-inflammatory analogues.

Moreover, recent advances in structure–activity relationship (SAR) and in silico modelling have provided valuable insights into the anti-inflammatory potential of flavonoids. SAR studies have highlighted that specific substitutions, such as hydroxylation patterns, prenylation, and methoxylation, can significantly influence bioactivity by modulating molecular interactions with key inflammatory targets such as COX-2, iNOS, and NF-κB [12,13]. Prenylation, for example, has been associated with enhanced membrane permeability and increased binding affinity due to improved lipophilicity [14].

Additionally, ADME prediction tools have been widely used to support the design and selection of new flavonoid analogues with optimized biological profiles. These computational approaches allow prediction of binding modes, interaction energies, and pharmacokinetic properties, complementing experimental data and guiding the rational development of more effective anti-inflammatory agents [15].

**Figure 1:**
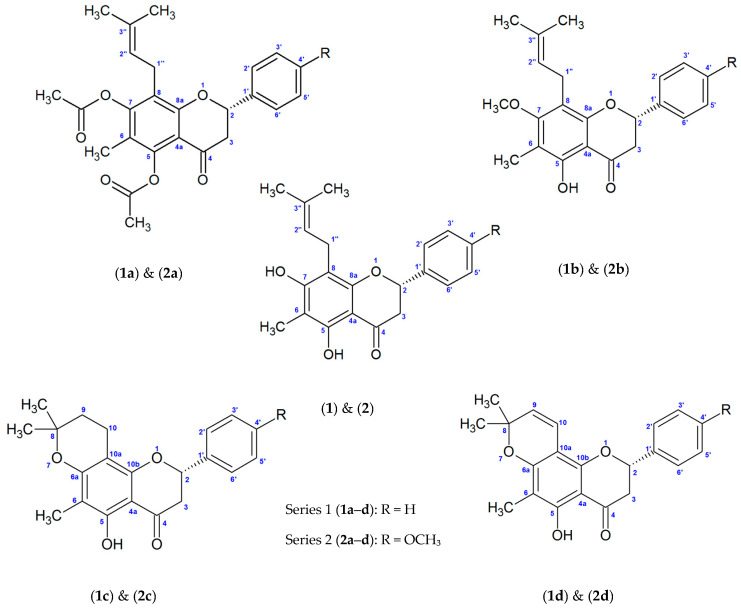
Chemical structure of natural flavanones (**1**) and (**2**) and their analogues (**1a**–**1d**) and (**2a**–**2d**).

Considering the potential anti-inflammatory of flavanones isolated from *E. platycarpa* leaves, in this study we describe the anti-inflammatory activity of natural flavanones (**1**) and (**2**) and of the analogues obtained from structural modifications (**1a**–**1d**) and (**2a**–**2d**) (Figure 1).

The objective of the present study was to evaluate of the anti-inflammatory activity of these flavonoid analogues (**1**, **1a**–**1d**) and (**2**, **2a**–**2d**) using an in vivo model of TPA-induced mouse ear edema. Additionally, physicochemical properties were characterized, and in silico and SAR analyses were performed to elucidate structure–activity relationships and support the identification of structural features relevant to their anti-inflammatory potential.

## 2. Results

### 2.1. Chemical Characterization

The natural flavanones (**1**) and (**2**) were isolated by column chromatography under reduced pressure, then purified and characterized by TLC in direct comparison with the original laboratory samples. Their identities were confirmed through ^1^H NMR, ^13^C NMR, and mass spectrometry, in agreement with the spectroscopic data previously reported by Narváez et al. [16].

Flavanone (**1**) (Figure 1) was obtained as a yellow solid (0.384 g, mp 174–177 °C) with a molecular ion at *m*/*z* 338, consistent with the molecular formula C_21_H_22_O_4_. Its IR spectrum showed characteristic bands at 3425.8 cm^−1^ (hydroxyl) and 1633 cm^−1^ (carbonyl).

Flavanone (**2**) (Figure 1) was isolated as a yellow solid (0.327 g, mp 152–155 °C) with a molecular ion at *m*/*z* 368, consistent with the molecular formula C_22_H_24_O_5_. Its IR spectrum displayed absorption at 3304.5 cm^−1^ (hydroxyl) and 1633.3 cm^−1^ (carbonyl).

Further structural elucidation of both compounds was achieved using advanced NMR techniques, including COSY, DEPT, HSQC, HMBC, and NOESY.

### 2.2. Pharmacomodulation

Pharmacomodulation has been a strategy used in Medicinal Chemistry for drug design, with the aim of improving the pharmacological potential. Once the lead compound is identified, a rational therapeutic strategy is developed to avoid the use of inactive compounds [17].

Moreover, the application of a consistent pharmacological or biochemical assessment method across a series of compounds is of considerable economic and scientific significance. This approach facilitates more accurate comparisons of activities and enables the development of more reliable structure–activity relationships [18]. By applying pharmacomodulation techniques, analogue compounds have been prepared from natural flavanones (**1**) and (**2**).

#### 2.2.1. Acetylation

Natural flavanones (**1**) and (**2**) were acetylated to generate the corresponding analogues (**1a**) and (**2a**), respectively [19]. Analogue (**1a**) was isolated as yellow solid in 80.5% yield, with a melting point of 68–70 °C, whereas analogue (**2a**) was isolated as yellow needle-shaped crystals in 79% yield, with a melting point of 73–75 °C. Structural confirmation was achieved by ^1^H NMR, ^13^C NMR, IR, and MS analyses.

Detailed experimental procedures, including reagents, reaction conditions, and purification methods, are provided in the Experimental Section.

#### 2.2.2. Methylation

Natural flavanones (**1**) and (**2**) were methylated to generate the corresponding analogues (**1b**) and (**2b**), respectively [20]. Analogue (**1b**) was isolated as a yellow solid in 77.5% yield, with a melting point of 108*–*111 °C, whereas analogue (**2b**) was obtained as a yellow oil in 58% yield. The structures of both methylated analogues were confirmed by ^1^H NMR, ^13^C NMR, IR, and MS analyses, consistent with the proposed *O*-methylated analogues.

#### 2.2.3. Cyclization

Natural flavanones (**1**) and (**2**) were subjected to intramolecular cyclization to generate the corresponding analogues (**1c**) and (**2c**), respectively. Analogue (**1c**) was isolated as a yellow solid, with melting point 51–53 °C, while analogue (**2c**) was obtained as a yellow oil.

Both cyclized products were purified by standard methods and fully characterized by ^1^H NMR, ^13^C NMR, IR, and MS analyses. The spectroscopic data confirmed the formation of the expected cyclized structures, demonstrating successful closure of the heterocyclic ring and the preservation of the core flavanone scaffold. These results are consistent with previously reported cyclization reactions of similar flavanone derivatives [8,21].

#### 2.2.4. Vinylogous-Cyclization

Natural flavanones (**1**) and (**2**) were subjected to vinylogous cyclization to generate the corresponding analogues (**1d**) and (**2d**), respectively [22].

Analogue (**1d**) was obtained as a yellow viscous liquid in 45% yield, while analogue (**2d**) was isolated as a yellow oil in 43.5% yield.

Both vinylogous-cyclized products were purified and fully characterized by ^1^H NMR, ^13^C NMR, IR, and MS analyses, confirming the formation of the new pyran ring and the expected structural modifications. Detailed experimental procedures are provided in the Experimental Section.

### 2.3. Physicochemical Property Determination

The physicochemical properties of the natural flavanones (**1**) and (**2**), their analogues (**1a**–**1d**) and (**2a**–**2d**) and Indomethacin were determined theoretically using the computational programme Hyperchem 8.0 to evaluate their drug-likeness and molecular behaviour. The physicochemical descriptors analyzed included: Total Energy (TE), Bond Energy (BE), Heat of Formation (H°f), frontier molecular orbital energies—HOMO (EHOMO) and LUMO (ELUMO)—Dipole Moment (µ), Molecular Surface Area (A), Molecular Weight (MW), Molecular Volume (MV), and Logarithm of the Partition Coefficient (Log P). The values of these electronic, steric, and lipophilic properties are summarized in Table 1.

### 2.4. In Silico Analyses

In silico prediction of ADME (Absorption, Distribution, Metabolism, and Excretion) properties was carried out using SwissADME. The evaluated parameters included gastrointestinal (GI) absorption, blood–brain barrier (BBB) permeability, Cytochrome P450 enzyme inhibition, topological polar surface area (TPSA), lipophilicity (Log P), and water solubility. These molecular descriptors provide valuable insights into the drug-likeness, oral bioavailability, and pharmacokinetic feasibility of the natural flavanones (**1**) and (**2**) and their analogues (**1a**–**1d**) and (**2a**–**2d**). The predicted values for these parameters are summarized in Table 2 and Table 3.

The predicted ADME profiles suggest that the structural modifications introduced, such as prenylation, methoxylation, and acetylation, influence key pharmacokinetic parameters, potentially enhancing the bioavailability and metabolic behaviour of these compounds.

In addition, the in silico studies allowed the prediction and evaluation of natural flavanones (**1**) and (**2**) and their analogues (**1a**–**1d**, **2a**–**2d**) prior to in vivo assays. The probability of each compound acting as an anti-inflammatory agent (Pa), estimated using the PASS Online programme, was generally around or above 0.7 for all compounds (Table 4), considering their predicted activity against specific proteins involved in inflammatory processes, including the following:

Together, these in silico predictions support the hypothesis that structural modifications introduced in the analogues (**1a**–**1d**) and (**2a**–**2d**) may enhance their interaction with inflammation-related molecular targets and modulate relevant biological activities. Therefore, the studied compounds emerge as promising candidates for further biological evaluation as potential selective anti-inflammatory agents.

### 2.5. In Vivo Anti-Inflammatory Activity Assay

The results obtained in the anti-inflammatory evaluation of natural flavanones (**1**) and (**2**) and their analogues (**1a**, **1b**, **1c**, **1d**, **2a**, **2b**, **2c** and **2d**) using the in vivo model of inflammation induction by TPA in the mouse ear are shown in Table 5. The dose of compounds in all cases was 1 mg/ear.

## 3. Discussion

### 3.1. Acetylation

**Figure 2:**
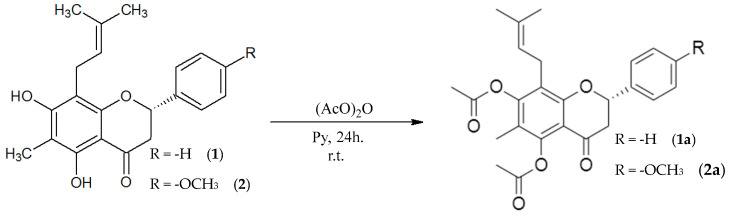
Acetylation reaction of natural flavanones (**1**) and (**2**) to obtain analogues (**1a**) and (**2a**).

Natural flavanones (**1**) and (**2**) were acetylated using acetic anhydride and pyridine, yielding analogues (**1a**) and (**2a**) [19]. The introduction of acetyl groups at positions 5 and 7 was confirmed by NMR analyses, in agreement with the structures proposed, these compounds are novel, and their nomenclature follows Andrade-Carrera et al. [8]. (Figure 2).

Acetylation is a well-established strategy in medicinal chemistry to modify hydroxyl groups, thereby increasing lipophilicity and improving the potential for membrane permeability, which is an essential property for drug-like candidates [18]. Previous reports have highlighted that flavonoids bearing hydroxyl, methoxy, or related substituents exhibit significant anti-inflammatory effects, often through suppression of pro-inflammatory mediators [23].

From a mechanistic perspective, acetylation proceeds through a nucleophilic substitution, where the hydroxyl groups of the flavanones attack the carbonyl carbon of acetic anhydride, resulting in the formation of acetyl esters. Pyridine facilitates this transformation by activating the hydroxyl groups and neutralizing acidic by-products [19].

The reactivity of the hydroxyl groups is also influenced by their electronic environment. In particular, the C-5 hydroxyl group is activated by intramolecular hydrogen bonding with the neighbouring C-4 carbonyl, which enhances its susceptibility to acetylation. This selectivity underscores the importance of local electronic effects in guiding semi-synthetic modifications of flavonoids [24,25].

### 3.2. Methylation

Methylation is a relevant strategy in medicinal chemistry because, by introducing methyl groups through the elimination of hydrogen and subsequent alkylation, the polarity of molecules is usually reduced, thereby increasing their lipophilicity. This structural modification can also affect recognition by endogenous and exogenous biomolecules through interactions with bioreceptors [20,26]. In general, the incorporation of one or more methyl groups into bioactive molecules tends to decrease water solubility while enhancing lipophilicity and promoting hydrophobic interactions, membrane permeability, and in some cases solubility itself, depending on the disruption of intramolecular hydrogen bonds, alteration of ionization states, or reduction in crystalline lattice energy [27]. These effects are often measured using the partition coefficient (Log P) between n-octanol and water [28].

**Figure 3:**
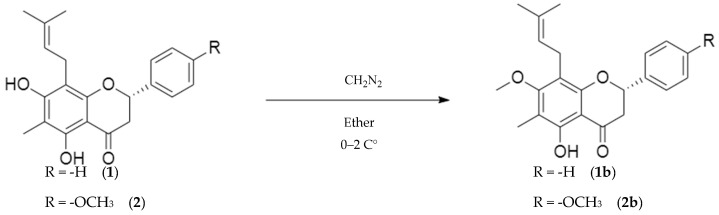
Reaction of methylation of natural flavanones (**1**) and (**2**) to produce analogues (**1b**) and (**2b**).

In the present study, natural flavanones (**1**) and (**2**) were methylated to yield the corresponding analogues (**1b**) and (**2b**) (Figure 3). The reaction followed a nucleophilic substitution mechanism (SN2), in which the phenolic hydroxyl groups attack the electrophilic carbon of diazomethane, producing *O*-methylated derivatives and releasing nitrogen gas (N_2_) as a by-product [29]. The ^1^H NMR spectra exhibited a singlet at 3.75 ppm corresponding to the methyl ether at C-7, while ^13^C NMR confirmed the methylation at 61.00 ppm for (**1b**) and 60.97 ppm for (**2b**). These compounds are novel, and their nomenclature followed Andrade-Carrera et al. [8].

The reactivity of hydroxyl groups in flavanones toward functionalization reactions such as acetylation and methylation is strongly influenced by the electronic characteristics of neighbouring substituents, particularly the carbonyl at C-4. Although both reactions involve nucleophilic substitution at hydroxyl groups, their mechanisms differ significantly. Acetylation is facilitated by the activation of the hydroxyl group at C-5 through chelation with the C-4 carbonyl, whereas methylation generally requires less electrophilic activation and does not benefit from such stabilizing interactions [29]. This difference explains why methylation of the 5-OH group was not considered in this study: the electronic environment enhances acetylation reactivity but not methylation [24,25]. Thus, acetylation proved to be a more favourable modification at that position, while methylation was restricted to other hydroxyl sites in the flavanones.

### 3.3. Cyclization

Cyclization is a widely used strategy in drug design, particularly valuable for studying the active conformation of flexible molecules. By restricting conformational freedom, this modification can stabilize the molecular scaffold, potentially improving selectivity and interaction with biological targets. However, it can also introduce new structural elements that may alter both biological activity and physicochemical properties, as well as generate new stereogenic centres [17].

**Figure 4:**
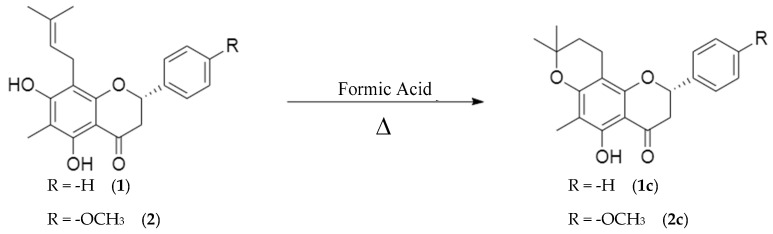
Reaction of cyclization of natural flavanones (**1**) and (**2**) to acquire analogues (**1c**) and (**2c**).

In this study, natural flavanones (**1**) and (**2**) were subjected to intramolecular cyclization to produce the corresponding pyrene analogues (**1c**) and (**2c**) (Figure 4). The reaction involves a nucleophilic attack of the hydroxyl group at position C-7 on the prenyl side chain at C-8, resulting in ring closure and formation of a new heterocyclic system. This transformation stabilizes the scaffold and introduces new structural features that can influence biological activity [8,30].

Analogue (**1c**) was isolated as a yellow solid, consistent with the compound previously reported by Ahluwalia in 1988, as confirmed by comparison of its physical and spectroscopic data [31]. Analogue (**2c**) was obtained as a yellow oil, displaying spectroscopic characteristics that confirmed its novel cyclized structure. In the ^1^H NMR spectrum, the hydrogens at C-1″ shifted from δ 3.32 ppm (doublet) in flavanone (**2**) to δ 2.60 ppm (doublet of triplets), consistent with coupling to H-2″α, H-2″β, and H-1″. The disappearance of the vinylic proton signal at δ 5.18 ppm (C-2″) and the appearance of a new triplet at δ 1.74 ppm further supported the cyclization. The ^13^C NMR spectrum confirmed the structural changes: C-1″ and C-2″appeared at δ 16.77 ppm and δ 32.04 ppm, respectively, consistent with methylene groups (verified by DEPT), while C-3″ appeared at δ 76.13 ppm, and the methyl groups shifted downfield to δ 26.84 and δ 27.58 ppm relative to the parent flavanone (**2**).

Taken together, these data demonstrate the formation of a cyclized analogue. Analogue (**2c**) is reported here for the first time, with nomenclature assigned according to Andrade-Carrera et al. [8].

### 3.4. Vinylogous-Cyclization

The principle of vinylogy can be explained as a consequence of resonance and has been successfully applied to conjugated systems, including imines, aromatic compounds, and alkynyl groups. However, the presence of vinyl groups introduces challenges due to their higher metabolic reactivity, which can lead to the formation of potentially toxic metabolites. Additionally, vinylogous cyclization can induce significant geometric and electronic changes, restricting conformational flexibility and increasing rigidity in cyclic systems [17].

**Figure 5:**
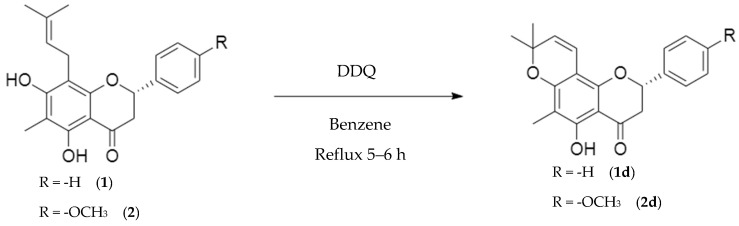
Reaction of vinylogous cyclization of natural flavanones (**1**) and (**2**) give analogues (**1d**) and (**2d**).

In this study, natural flavanones (**1**) and (**2**) were subjected to intramolecular vinylogous cyclization to yield the corresponding analogues (**1d**) and (**2d**) (Figure 5). The reaction was performed using 2,3-dichloro-5,6-dicyanobenzoquinone (DDQ) under rigorously anhydrous conditions, following the methodology reported by Nagar et al. [22]. After recrystallization, the products were obtained as yellow viscous liquids.

Mechanistically, vinylogous cyclization involves a π-electron delocalization-driven intramolecular attack, in which the electron-rich hydroxyl group interacts with the conjugated double bond of the prenyl substituent. This leads to the formation of a new pyran ring, restricts conformational flexibility, and changes the hybridization of carbons C-9 and C-10 from sp^3^ to sp^2^, altering the electronic distribution of the system.

The structures of analogues (**1d**) and (**2d**) were confirmed by spectroscopic analysis. IR spectra displayed characteristic bands for hydroxyl groups, ketone carbonyls, and aromatic C=C bonds, consistent with the proposed structures. In the ^1^H NMR spectrum, analogue (**1d**) showed doublets at δ 6.60 ppm and δ 5.47 ppm, corresponding to vinylogous protons H-1″ and H-2″, respectively. Analogue (**2d**) exhibited similar doublets at δ 6.55 ppm (H-1″) and δ 5.46 ppm (H-2″). ^13^C NMR analysis confirmed the vinylogous carbons: C-1″ and C-2″ resonated at δ 115.77 ppm and δ 126.09 ppm for analogue (**1d**), and at δ 116.10 ppm and δ 127.68 ppm for analogue (**2d**).

Taken together, these data demonstrate the successful formation of novel vinylogously cyclized derivative. Analogue (**2d**) is reported here for the first time in the literature, with nomenclature assigned according to Andrade-Carrera et al. [8].

Detailed proposed mechanisms for all the reactions discussed above are provided in the Supplementary Material (Scheme S1).

### 3.5. Structure–Activity Relationship (SAR), Drug-likeness and Biological Implications

The physicochemical and in silico analyses of natural flavanones (**1**) and (**2**) and their analogues (**1a**–**1d**) and (**2a**–**2d**) revealed a clear influence of structural modifications on their molecular behaviour, drug-likeness, and predicted biological activity. Computational descriptors derived from HyperChem and SwissADME, along with PASS online predictions.

The analogues (**1a**–**1d**) and (**2a**–**2d**) exhibited lower total energy (TE) and bond energy (BE) values than natural flavanones (**1** and **2**), indicating higher structural stability and greater thermodynamic favourability, especially in analogues (**1a**) and (**2a**). These observations align with literature suggesting that lower TE is associated with enhanced molecular rigidity, potentially contributing to greater target selectivity [32].

In addition, the heat of formation (H°f) values were also more negative for analogues, particularly (**2a**) (−213.6 kcal/mol), suggesting that the incorporation of substituents such as methoxy and prenyl groups enhances thermodynamic favourability. These modifications likely contribute to improved π-conjugation and hydrogen bonding potential, which are crucial for biological activity [33].

The dipole moment (µ), an important factor in determining intermolecular interactions, solubility and biological target recognition, was highest for analogue (**2c**) (4.93 D), suggesting a higher degree of polarity and interaction potential [34]. Additionally, frontier molecular orbital analysis showed that analogues (**1c**) and (**2c**) had slightly narrower HOMO–LUMO gaps, which correlates with increased electronic reactivity and potential for biological activity [35].

From a drug-likeness perspective, all compounds satisfied the criteria defined by Lipinski’s Rule of Five, indicating potential for good oral bioavailability [36]. Particular attention was given to the logarithm of the partition coefficient (Log P), a critical descriptor of lipophilicity. Two computational approaches were used, HyperChem and Swiss Target platform, revealing differences due to methodological variability.

According to HyperChem, Log P values ranged from −1.43 (**2d**) to 0.88 (**1b**), suggesting a predominantly hydrophilic profile. Conversely, Swiss Target-based predictions ranged from 2.67 (**2d**) to 4.46 (**1a**), suggesting greater lipophilic character. This discrepancy is expected, as each tool relies on distinct predictive algorithms and training data. Notably, analogue (**1a**) displayed the highest lipophilicity (Log P = 4.46), approaching the Lipinski threshold (Log P ≤ 5), implying favourable membrane permeability but necessitating monitoring of aqueous solubility. Meanwhile, analogue (**2d**), with the lowest Log P in both models, may exhibit improved water solubility, beneficial for absorption but potentially limiting in terms of membrane permeability [37].

Overall, the range of Log P values across the series indicates a favourable balance between hydrophilicity and lipophilicity, which is desirable during lead optimization. Compounds with Log P values around 2 to 3 are often ideal for oral drug candidates due to a suitable balance between permeability and solubility.

In the in vivo anti-inflammatory evaluation using the TPA-induced mouse ear edema model, several compounds demonstrated potent activity, particularly analogues (**2c**) (98.62%), (**2d**) (76.12%), and (**1c**) (71.64%). These inhibition percentages were comparable or even superior to the reference drug Indomethacin (91.00%), highlighting their strong therapeutic potential.

The high efficacy of these compounds can be linked to their computationally predicted physicochemical properties. For instance, analogue (**2c**), with a high dipole moment (4.93 D), moderately low H°f (−159.67 kcal/mol), and Log P of −0.79 (HyperChem), demonstrated a favourable profile for interaction with polar biological targets, topical bioavailability, and aqueous solubility. These features are frequently associated with improved biological responses in flavonoid-based anti-inflammatory agents [18,38].

Moreover, the electronic reactivity of analogue (**2c**), as suggested by its HOMO (−8.751 eV) and LUMO (−0.389 eV) energies, indicates potential for redox interactions with enzymatic targets such as cyclooxygenase-2 (COX-2) and 5-lipoxygenase (5-LOX), both of which play pivotal roles in inflammatory processes [39,40].

Structurally, the chemical modifications introduced in the analogues, particularly methoxylation and prenylation, proved essential in enhancing anti-inflammatory activity. These groups modulated molecular polarity, size, and electronic properties without compromising drug-likeness. This is consistent with previous studies demonstrating that strategic substitutions on flavonoid scaffolds can significantly improve both biological efficacy and pharmacokinetics [41].

In contrast, natural flavanone (**1**) (12.20%) and its analogue (**1a**) (16.02%) showed poor anti-inflammatory activity, which may be attributed to lower dipole moments, higher formation energies, and suboptimal Log P values. These characteristics likely result in reduced interaction with biological targets or poor membrane permeability, echoing previous observations on the limitations of unmodified natural products in drug development [42].

#### 3.5.1. ADME Properties and Predicted Drug Behaviour

SwissADME analysis demonstrated that all compounds (**1**, **2**, **1a**–**1d**, **2a**–**2d**) exhibited high gastrointestinal absorption, indicating potential for oral administration. Notably, analogues like (**1c**, **2b**, and **2c**) were also predicted to cross the blood–brain barrier (BBB), indicating possible central anti-inflammatory activity. Conversely, analogues such as (**1a**) and (**2a**) lacked BBB permeability, which could be beneficial in avoiding central nervous system (CNS)-related side effects during peripheral inflammation treatment [43].

Topological polar surface area (TPSA) values for all compounds were under 90 Å^2^, supporting good passive diffusion across biological membranes [44].

With respect to metabolic predictions, several compounds were forecasted to inhibit cytochrome P450 isoforms, particularly CYP3A4 and CYP2C9. While this raises concerns regarding potential drug–drug interactions, it could also imply a longer half-life and prolonged therapeutic effect, though this would require confirmation via in vivo pharmacokinetics [45].

Water solubility predictions (Log S) indicated moderate solubility for analogues such as (**2d**) (Log S = −3.88), supporting ease of formulation. In contrast, poorly soluble analogues like (**2b**) (Log S = −5.56) may require solubilization strategies, such as encapsulation or co-solvent systems, to enhance bioavailability [46].

#### 3.5.2. Predicted Biological Activity via PASS Online

PASS Online analysis supported the anti-inflammatory and antioxidant potential of the analogues, especially analogues (**1b**), (**1c**), (**2b**), and (**2c**), which exhibited high Pa values (>0.9) in several relevant biological activities. These included lipid peroxidase inhibition, free radical scavenging, and NOS2 expression inhibition, suggesting a multi-target mechanism involving both oxidative stress mitigation and inflammatory mediator suppression.

Analogue (**2c**) emerged as particularly promising, showing the following Pa values; NOS2 expression inhibition (0.806); Free radical scavenging (0.858) and Lipid peroxidase inhibition (0.943).

These functions are crucial, as NOS2 (inducible nitric oxide synthase) is a pro-inflammatory enzyme whose overexpression leads to nitric oxide–mediated tissue damage. Likewise, inhibition of lipid peroxidation can prevent membrane destabilization and interrupt the inflammatory cascade [47,48].

Analogue (**1c**), which also showed strong in vivo efficacy, shared a similar profile, e.g., lipid peroxidase inhibition: Pa = 0.930, reinforcing the central role of antioxidant activity in the overall anti-inflammatory effect.

Interestingly, while indomethacin showed strong in vivo efficacy (91.00%), its predicted PASS activities in antioxidant-related pathways were significantly lower (e.g., free radical scavenging: Pa = 0.151; cytoprotection: Pa = 0.285). This suggests that indomethacin acts primarily through COX inhibition, while the analogues may achieve broader therapeutic benefits through multi-pathway modulation, which is particularly valuable in chronic inflammatory conditions [49].

#### 3.5.3. Structure–Activity Relationships and In Silico–In Vivo Correlation

There was a strong correlation between the in silico predictions and in vivo anti-inflammatory activity, validating the predictive power of the computational models. The most active analogues are (**2c**) (98.62%), (**2d**) (76.12%), and (**1c**) (71.64%), also exhibited favourable pharmacokinetic and physicochemical profiles, moderate lipophilicity (Log P < 4), high gastrointestinal absorption, supporting oral delivery, optimal TPSA (~65 Å^2^) for membrane transport and high Pa values for antioxidant and anti-inflammatory bioactivities.

In contrast, natural flavanone (**1**) (12.2%) and analogue (**1a**) (16.02%) showed lower Pa values, unfavourable ADME predictions, and poor in vivo efficacy, underscoring the need for a multi-parameter optimization strategy. The combined analysis supports the rationale that compounds with balanced physicochemical properties, favourable pharmacokinetics, and multi-target mechanisms are more likely to be successful anti-inflammatory agents.

Ultimately, this study highlights the utility of integrated in silico platforms, including SwissADME and PASS Online, in guiding the rational design of flavonoid-based drug candidates. Compounds like (**2c**) and (**1c**), with both predictive and experimental validation, exemplify how multi-target engagement may offer superior efficacy and reduced side effects compared to single-target NSAIDs [50].

### 3.6. Anti-Inflammatory Assay

Indomethacin, a well-established nonsteroidal anti-inflammatory drug (NSAID), was used as a positive control in this study and demonstrated an inhibition percentage of 91.00 ± 0.46%. When compared to indomethacin, the natural flavanone (**1**) showed the lowest anti-inflammatory activity (12.20 ± 1.12%), indicating the potential for improvement through structural modifications. It was observed that the anti-inflammatory activity increased with respect to the structural modifications of the natural flavonoids. Notably, analogue (**2c**), a cyclized derivative, exhibited a significantly higher anti-inflammatory effect, with an inhibition percentage of 98.62 ± 1.92%. This enhanced activity suggests that the rigidity introduced by cyclization may favour stronger interactions with key therapeutic targets involved in inflammation, such as cyclooxygenases (COXs). COX enzymes, particularly COX-1 and COX-2, are critical mediators in the inflammatory process through the production of prostaglandins [51]. Rigidified compounds like analogue (**2c**) may improve binding to the COX active site due to their conformational stability, similar to the known mechanism of action of indomethacin [52]. In contrast, analogue (**1c**), which lacks the methoxy group on the C ring, showed a lower anti-inflammatory activity (71.64 ± 1.86%). The presence of the methoxy group in analogue (**2c**) likely contributes to increased permeability and enhanced tissue distribution, as methoxy groups are known to improve the lipophilicity of compounds [53]. These results suggest that while molecular rigidity plays a crucial role in enhancing interactions with COXs, the methoxy group may further optimize the pharmacokinetic profile of the compound, contributing to its superior anti-inflammatory efficacy.

On the other hand, for the other cyclized analogue (**2d**) exhibits an anti-inflammatory activity of 76.12 ± 1.74%, and when compared to analogue (**1d**), a difference of 38% is observed, thereby indicating the effect of the methoxy group.

The activity between analogues (**2c**) and (**2d**) represents a decrease in analogue (**2d**) with a difference of 22.5% and the only difference is the presence of a conjugated double bond, and this characteristic makes the compound more reactive and can generate toxic metabolites.

Regarding analogues (**1c**), (**2c**) and (**2d**) their anti-inflammatory activity was higher compared to natural flavanones (**1**) and (**2**), respectively, observing that the structural change in the hydroxyl in position 7 by another radical of greater size favours the increase in its activity.

Statistical analysis demonstrated that several analogues (especially (**1c**) and (**2c**) were significantly more active than their natural flavanones (**1**) and (**2**), supporting the strategy of semisynthetic optimization to improve efficacy. These results are consistent with other studies that have shown prenylated flavonoids possess increased lipophilicity and cell membrane affinity, enhancing their anti-inflammatory activity [54].

#### Anti-Inflammatory Mechanism and Medicinal Chemistry Implications

As shown in Section 3.5, analogue (**2c**) exhibited the highest in vivo anti-inflammatory activity (98.62%), markedly surpassing both the natural flavanones (**1**) and (**2**) and other analogues. This enhanced activity can be rationalized through structural and mechanistic considerations. The cyclization of the prenyl group introduces conformational rigidity, which is known to enhance binding affinity and selectivity toward molecular targets such as COX-2 and iNOS) [11,55]. Rigidified flavanones reduce conformational entropy penalties upon binding, favouring stable enzyme–ligand complexes compared to more flexible natural flavanones (Section 3.4 and Section 3.5).

The methoxy substituent at position 4′ further contributes to activity by increasing lipophilicity, cellular uptake, and potentially metabolic stability (Section 3.4) [56]. In analogue (**2c**), the cyclization of the prenyl group introduces conformational rigidity rather than free prenyl interactions, thereby reducing entropic penalties upon binding and favouring more stable enzyme–ligand complexes. The combined effect of structural rigidification and methoxylation synergistically optimizes both pharmacodynamic and pharmacokinetic profiles.

From a mechanistic perspective, flavanones are known to exert anti-inflammatory effects Via:

- Enzyme inhibition: COX-2 and iNOS inhibition, reducing prostaglandin E2 (PGE2) and nitric oxide (NO) production.
- Signalling modulation: Suppression of NF-κB signalling, leading to decreased production of pro-inflammatory cytokines such as TNF-α, IL-1β, and IL-6 [57,58]. Analogue (**2c**), with its rigidified structure and methoxy group, likely stabilizes interactions at enzyme active sites and with regulatory proteins involved in cytokine expression (Section 3.5).

A summary of the structure–activity relationship (SAR) observed in this study is presented in Table 6:

These observations are in line with previous reports showing that structurally rigidified flavanones, particularly those obtained through prenyl-derived cyclization, exhibit superior anti-inflammatory activity compared to unsubstituted flavanones, both in vitro and in vivo [11,55,59].

From a medicinal chemistry standpoint, analogue (**2c**) offers several advantages:

1. Enhanced target binding and selectivity due to structural rigidification introduced by cyclization.
2. Improved pharmacokinetics, with the methoxy substituent enhancing lipophilicity, membrane permeability, and potential metabolic stability [56].
3. Retention of the flavanone core scaffold, preserving antioxidant properties and a favourable safety profile.
4. Lead optimization potential for semi-synthetic derivatives to further improve efficacy and bioavailability in anti-inflammatory drug development.

Importantly, the slight decrease in activity observed in analogue (**2d**) compared to (**2c**) highlights that even subtle structural changes, such as the presence of a conjugated double bond, can impact reactivity and potential for metabolite formation, emphasizing the importance of precise SAR-guided design (Section 3.5).

Overall, analogue (**2c**) exemplifies a strategically optimized flavanone derivative, in which rigidification and methoxylation collectively enhance anti-inflammatory efficacy and provide a favourable platform for further medicinal chemistry development.

## 4. Materials and Methods

### 4.1. Plant Material

Leaves of *E. platycarpa* Pennell & Safford (*Fabaceae*) were collected in Tetipac, Guerrero, Mexico, (GPS: 18°33′58″ N, 99°36′45″ W). Voucher specimens (No. 1325) were authenticated by Prof. Ramiro Cruz Durán and deposited at Herbarium of the Faculty of Sciences, Universidad Nacional Autónoma de México.

### 4.2. Materials and Instrumentation

Solvents, deuterated solvents, and reagents were obtained from Sigma-Aldrich (Toluca de Lerdo, Mexico). FTIR spectra were recorded on a Bruker Vector 22 spectrometer (Thermo Electron Scientific, Madison, WI, USA) over 525–4000 cm^−1^, using a KBr beam splitter, a DTGS detector, and OMNIC^®^ software version 9.2. ^1^H and ^13^C NMR spectra were acquired on a spectra were recorded in a Unity NMR spectrometer (Varian Inova, Palo Alto, CA, USA) operating at 400 MHz for ^1^H and 200 MHz for ^13^C nuclei with 15 mg of each compound dissolved in CDCl_3_ and TMS as internal standard. Molecular formulas of compounds (**1a**–**1d**) and (**2a**–**2d**) were determined by fast atom bombardment mass spectrometry (FABMS) using an Agilent 6890 GC (Agilent Technologies, Santa Clara, CA, USA) coupled to an Agilent 5973N mass spectrometer. High-resolution mass spectra were obtained using an MStation JMS-700 (JEOL, Peabody, MA, USA). (M^+^H^+^) and data mass calculated was obtained from ChemSketch (ACD/Labs, version 12.0, Toronto, ON, Canada).

### 4.3. Preparation of Methanolic Extract

Shade-dried leaves of *E. platycarpa* were pulverized and subjected to triplicate maceration in methanol at room temperature (100 g of dried material per 1000 mL of solvent). The combined extracts were filtered and concentrated under reduced pressure to afford 166.3 mg of dry extract per gram of dried leaves [16].

### 4.4. In Silico Physicochemical and ADME Analysis

The physicochemical properties of natural flavanones (**1**) and (**2**) and their analogues (**1a**–**1d**) and (**2a**–**2d**) were calculated using HyperChem 8.0 (Hypercube Inc., Gainesville, FL, USA). The computer system used in this study was equipped with an Acer 3 laptop (Acer Inc., New Taipei City, Taiwan), equipped with an Intel Core i3-1005G1 CPU processor, 12 GB RAM, and Windows 11 operating system. The geometry of all compounds was initially optimized using the MM+ force field and subsequently used as input structures for AM1 semiempirical calculations. Energy minimization was performed using the Polak-Ribiere algorithm until a maximum gradient of 0.10 kcal/(Å·mol) or 1485 maximum cycles was reached in vacuum.

Pharmacokinetic parameters and ADME properties (absorption, distribution, metabolism, and excretion) were predicted using the SwissADME online platform (http://www.swissadme.ch, accessed on 1 March 2025). Potential biological activities and toxicity risks were assessed using PASS online (https://way2drug.com/PassOnline/index.php, accessed on 9 March 2025), with probability values (Pa > 0.5) considered indicative of potential activity [60].

### 4.5. Anti-Inflammatory Activity

#### 4.5.1. Experimental Animals

Male CD1 mice (body weight range 22–25 g) were used and maintained under standard Bioterio conditions, with free access to food and water, on a 12 h light–dark cycle. Euthanasia was performed in accordance with the guidelines established by the Official Mexican Standard NOM-062-ZOO-1999 [61].

#### 4.5.2. TPA-Induced Mouse Ear Edema

The in vivo TPA-induced mouse ear edema assay was performed as previously described by Domínguez-Villegas, V. et al. Briefly, groups of three male mice received a topical application of 2.5 μg TPA per ear in ethanol. Natural flavanones (**1**) and (**2**) and their analogues (**1a**–**1d**) and (**2a**–**2d**) and indomethacin (used as a positive control) were applied at 1 mg per ear. All treatments were dissolved in ethanol and applied topically on both ears. Each of the compounds was administered topically in the right ear such as TPA. The swelling ear was evaluated after 6 h by comparison with a group of mice applying only the vehicles without inhibitor (negative control). The percentage of inflammation (%) for each mouse was calculated by the following formula, Equation (1):

(1) $$\%Inhibition=\frac{∆Wc-∆We}{∆Wc}\;\times \;100$$

where:

ΔWc: weight of the negative control auricular tissue sections;
ΔWe: weight of tested compound.

### 4.6. Isolation of Natural Flavanones (1) and (2)

Prenylated flavanones were isolated from the methanolic extract of *E. platycarpa* leaves by column chromatography under reduced pressure using silica gel 60. Flavanone (**1**) [(2*S*)-5,7-dihydroxy-6-methyl-8-(3-methyl-2-buten-1-yl)-2-phenyl-2,3-dihydro-4*H*-1-benzopyran-4-one, 121 mg] and flavanone (**2**) [(2*S*)-5,7-dihydroxy-2-(4′-methoxyphenyl)-6-methyl-8-(3-methyl-2-buten-1-yl)-2,3-dihydro-4*H*-1-benzopyran-4-one, 118 mg] were obtained as yellow solids. Both compounds were purified, characterized by TLC against the original samples, and confirmed by ^1^H- and ^13^C-NMR, as well as mass spectrometry, with identities verified through comparison with previously published spectroscopic data [16].

### 4.7. Preparation of Analogues (***1a***–***1d***) and (***2a***–***2d***)

#### 4.7.1. Preparation of Acetylated Analogues (**1a**) and (**2a**)

A solution of natural flavanone (**1**, 20.00 mg, 0.0591 mmol) or (**2**, 20.00 mg, 0.0543 mmol) in acetone (2 mL) was reacted with an acetic anhydride–pyridine mixture (2:1, 4 mL) at room temperature for 24 h. After standard work-up, the crude products were purified by preparative TLC using n-hexane: EtOAc (9:1) to afford the acetylated analogues [19].

Acetylated analogue (**1a**):

(2*S*)-5,7-bis(acetyloxy)-6-methyl-8-(3-methyl-2-buten-1-yl)-2-phenyl-2,3-dihydro-4*H*-1-benzopyran-4-one.

^1^H-NMR (400 MHz, chloroform-d, δ; ppm)

5.44 dd, 12.8, 3.2 Hz, H-2; 3.03 dd, 16.8, 12.8 Hz, H-3α; 2.87 dd, 16.8, 3.2 Hz; H-3β; 2.03 s, 6 (-CH_3_); 7.44 m, -C_6_H_5_; 3.02 s, H-1″; 4.89 tc, 6.8, 1.2 Hz, H-2″; 1.77 s, 3″(-CH_3_), 1.67 s, 3″(-CH_3_); 2.03 s, 5 (-OCOCH_3_); 2.33 s, 7 (-OCOCH_3_).

^13^C-NMR (100 MHz, Chloroform-d, δ; ppm)

79.32 C-2; 45.50 C-3; 198.18 C-4; 106.56 C-4a; 156.86 C-5; 128.86 C-6; 10.03, 6(-CH_3_); 161.33 C-7; 106.78 C-8; 159.67 C-8a; 138.67 C-1′; 126.03 C-2′/C-6′; 128.94 C-3′/C-5′; 128.94 C-4′; 23.84 C-1″; 121.31 C-2″; 132.33 C-3″; 25.88 3″(-CH_3_); 18.16 3″(CH_3_); 169.11, 5-(-OCOCH_3_); 21.33, 5 (-OCOCH_3_) 166.95, 7-(-OCOCH_3_); 20.57, 7 (-OCOCH_3_).

IR; 2921.8 cm^−1^, 2371.9 cm^−1^, 1770.6 cm^−1^, 1688.7 cm^−1^, 1608.0 cm^−1^, 1451.5 cm^−1^, 1370.5 cm^−1^, 1283.0 cm^−1^, 1193.5 cm^−1^, 1155.3 cm^−1^, 1107.0 cm^−1^, 1005.9 cm^−1^, 895.9 cm^−1^, 764.6 cm^−1^, 699.3 cm^−1^, 577.8 cm^−1^.

HR-MS (ESI), calcd. for C_25_H_26_O_6_: [M^+^H^+^] 423.1729, found: 423.1811 ([M^+^H^+^]).
Acetylated analogue (**2a**):

(2*S*)-5,7-bis(acetyloxy)-2-(4′-methoxyphenyl)-6-methyl-8-(3-methyl-2-buten-1-yl)-2,3-dihydro-4*H*-1-benzopyran-4-one

^1^H-NMR (400 MHz, chloroform-d, δ; ppm)

5.42 dd, 12.8, 2.70 Hz, H-2; 2.73 dd, 14.17, 10.19 H-3α; 2.4 dd, 14.17, 2.47, H-3β; 2.33 s, 6(-CH3); 7.37 ddd, 6.90, 1.5, Hz, H-2′/H-6′; 6.95 ddd, 6.9, 1.5, Hz, H-3′/H-5′; 3.84 s, 4′(-OCH_3_); 7.30 d, 7.3 H-1″; 4.98 t, 7.3, Hz, H-2″; 1.72 s, 3″(-CH_3_); 1.66 s, 3″(-CH_3_); 2.01 s, 5 (-OCOCH_3_); 2.02 s, 7 (-OCOCH_3_).

^13^C-NMR (100 MHz, chloroform-d, δ; ppm)

79.11 C-2; 45.38 C-3; 198.18 C-4; 114.31 C-4a; 153.55 C-5; 9.90, C-6; 9.04 6(-CH_3_); 140.6 C-7; 111.89 C-8; 159.99 C-8a; 138.12 C-1′; 127.64 C-2′/C-6′; 130.74 C-3′/C-5′; 146.55 C-4′; 55.53 4′(-OCH_3_); 23.73 C-1″; 121.39 C-2″; 132.26 C-3″; 21.24, 3″(CH_3_); 20.66, 3″(CH_3_); 169.54, 5-(-OCOCH_3_); 18.04, 5 (-OCOCH_3_) 167.98, 7 (-OCOCH_3_); 18.04, 7 (-OCOCH_3_).

IR; 2932.3 cm^−1^, 1636.8 cm^−1^, 1576.8 cm^−1^, 1446.4 cm^−1^, 1383.6 cm^−1^, 1347.9 cm^−1^, 1299.0 cm^−1^, 1242.6 cm^−1^, 1164.4 cm^−1^, 1100.2 cm^−1^, 883.7 cm^−1^, 829.2 cm^−1^, 771.3 cm^−1^, 699.9 cm^−1^, 603.1 cm^−1^, 474.3 cm^−1^.

HR-MS (ESI), calcd. for C_26_H_28_O_7_: [M^+^H^+^] 453.1835, found: 453.1795 ([M^+^H^+^]).

#### 4.7.2. Preparation of Methylated Analogues (**1b**) and (**2b**)

Natural flavanones (**1**, 20 mg; 0.0591 mmol) and (**2**, 20 mg; 0.0543 mmol) were dissolved in a mixture of absolute ethanol and ethyl ether. The reaction mixture was treated dropwise with an ethereal solution of diazomethane, prepared from nitromethyl urea (2.8 g), while cooling in an ice bath for 10 min. During addition, the reaction mixture turned pale yellow. The mixture was then maintained in an ice chest for 5 h with a vented closure to allow gas release [20].

After complete solvent removal, the residue was concentrated, cooled in an ice–water bath, and the resulting methyl ether was allowed to crystallize. The methylated analogues were purified by recrystallization.

Methylated analogue (**1b**):

(2*S*)-5-hydroxy-7-methoxy-6-methyl-8-(3-methyl-2-buten-1-yl)-2-phenyl-2,3-dihydro-4*H*-1-benzopyran-4-one.

^1^H-NMR (400 MHz, chloroform-d, δ; ppm)

5.42 dd, 12.8, 3.2 Hz, H-2; 3.05 dd, 17.2, 12.8 Hz, H-3α; 2.86 dd, 17.2, 3.2, H-3β; 12.026 s, 5 (-OH); 2.101 s, 6 (-CH_3_); 7.45 m, -C_6_H_5_; 3.31 d, 6.8 Hz, H-1″; 5.21 tc, 6.8, 1.2 Hz, H-2″;1.791 s, 3″-CH_3_; 1.69 s, 3″-CH_3_; 1.69 s, 3″-CH_3_, 3.75 s, 7 (-OCH_3_).

^13^C-NMR (100 MHz, Chloroform-d, δ; ppm)

78.76 C-2; 43.97 C-3; 197.34 C-4; 105.35 C-4a; 159.21 C-5; 115.59 C-6; 165.37 C-7; 109.94 C-8; 158.23 C-8a; 138.93 C-1′; 126.00 C-2′/C-6′; 128.90 C-3′/C-5′; 128.9 C-4′; 22.37 C-1″; 122.87 C-2″; 131.81 C-3″; 18.16 3″(CH_3_); 26.05 3″(CH_3_).

IR; 3440.0 cm^−1^, 3031.0 cm^−1^, 2922.0 cm^−1^, 2854.0 cm^−1^, 2384.0 cm^−1^, 1627.0 cm^−1^, 1586.0 cm^−1^, 1441.4 cm^−1^, 1369.7 cm^−1^, 1299.6 cm^−1^, 1162.6 cm^−1^, 1119.3 cm^−1^, 970.3 cm^−1^, 902.8 cm^−1^, 762.5 cm^−1^, 697.9 cm^−1^, 638.0 cm^−1^, 556.8 cm^−1^.

HR-MS (ESI), calcd. for C_22_H_24_O_4_: [M^+^H^+^] 353.1674, found: 353.1720 ([M^+^H^+^]).
Methylated analogue (**2b**):

(2*S*)-5-hydroxy-7-methoxy-2-(4′-methoxyphenyl)-6-methyl-8-(3-methyl-2-buten-1-yl)-2,3-dihydro-4*H*-1-benzopyran-4-one.

^1^H-NMR (400 MHz, chloroform-d, δ; ppm)

5.35 dd, 12.8, 3.2 Hz, H-2; 3.08 dd, 17.2, 12.8 Hz, H-3α; 2.83 dd, 17.2, 3.2 Hz, H-3β; 12.08 s, 5 (-OH); 2.1 s, 6(-CH_3_); 7.39 d, 8.8 Hz, H-2′/H-6′:6.94 d, 8.8 Hz, H-3′/H-5′; 3.84 s, 4′(-OCH_3_); 3.30 d, 7.2 Hz, H-1″, 5.2 t, 7.2 Hz, H-2″; 1.65 s, 3″(-CH_3_); 1.62 s, 3″(-CH_3_); 3.5 s 7 (-OCH_3_).

^13^C-NMR (100 MHz, chloroform-d, δ; ppm)

78.65 C-2; 43.77 C-3; 197.59 C-4; 105.32 C-4a; 159.67 C-5; 105.62 C-6; 8.48 6(-CH_3_); 165.31 C-7; 109.89 C-8; 157.89 C-8a; 130.95 C-1′; 127.61 C-2′/C-6′; 114.25 C-3′/C-5′; 158.35 C-4′; 55.58 4′(-OCH_3_); 21.32 C-1″, 123.119 C-2″; 130.95 C-3″; 26.07 3″(-CH_3_); 18.104 3″(-CH_3_).

IR; 3438.1 cm^−1^, 2924.4 cm^−1^, 2857.0 cm^−1^, 2372.7 cm^−1^, 2331.0 cm^−1^, 1630.9 cm^−1^, 1515.9 cm^−1^, 1458.4 cm^−1^, 1250.7 cm^−1^, 1120.2 cm^−1^, 948.2 cm^−1^, 466.0 cm^−1^.

HR-MS (ESI), calcd. for C_23_H_26_O_5_: [M^+^H^+^] 383.1780, found: 383.1801 ([M^+^H^+^]).

#### 4.7.3. Preparation of Cyclized Analogues (**1c**) and (**2c**)

Natural flavanones (**1**, 20 mg, 0.0591 mmol) and (**2**, 20 mg, 0.0543 mmol) were refluxed in formic acid (10 mL) at 60 °C for 2 h, and then cooled to room temperature for 6 h. The reaction mixture was recrystallized from ice–water, extracted with ethyl acetate, washed three times with water, and dried over anhydrous Na_2_SO_4_ [62].

Cyclized analogue (**1c**):

(2*S*)-5-hydroxy-6,8,8-trimethyl-2-phenyl-2,3,9,10-tetrahydro-4*H*,8*H*-benzo [1,2-*b*:3,4-*b′*]dipyran-4-one.

^1^H-NMR (400 MHz, chloroform-d, δ; ppm)

5.42 dd, 12.8, 3.2 Hz, H-2; 3.02 dd, 17.2, 12.8 H-3α; 2.83 dd, 17.2, 3.2, H-3β; 12.33 s, 5 (OH); 1.98 s, 6 (-CH_3_); 7.43 m, -C_6_H_5_; 2.64 td, 7.2, 3.2 Hz, H-1″; 1.79 t,6.8 Hz, H-2″; 1.35 s, 3″(-CH_3_); 1.25 s, 3″(-CH_3_).

^13^C-NMR (100 MHz, chloroform-d, δ; ppm)

78.60 C-2; 43.76 C-3; 196.25 C-4; 101.79C-4a; 159.23 C-5; 102.18 C-6; 7.766 (-CH_3_); 161.20 C-7; 104.51 C-8; 157.37 C-8a; 139.44 C-1′; 126.13 C-2′/C-6; 128.95 C-3′/C-5′; 128.95 C-4′; 27.01 C-1″; 126.13 C-2″; 128.95 C-3″, 16.18 3″(-CH_3_); 27.22 3″(CH_3_).

IR; 3753 cm^−1^, 3448 cm^−1^, 2921 cm^−1^, 2371 cm^−1^, 1637 cm^−1^, 1447 cm^−1^, 1376 cm^−1^, 1305 cm^−1^, 1168 cm^−1^, 1119 cm^−1^, 899 cm^−1^, 805 cm^−1^, 758 cm^−1^, 694 cm^−1^, 607 cm^−1^, 479 cm^−1^.

HR-MS (ESI) (**1c**), calcd. for C_21_H_22_O_4_: [M^+^H^+^] 339.1518, found: 329.1616 ([M^+^H^+^]).
Cyclized analogue (**2c**):

(2*S*)-5-hydroxy-2-(4′-methoxyphenyl)-6,8,8-trimethyl-2-phenyl-2,3,9,10-tetrahydro-4*H*,8*H*-benzo [1,2-*b*:3,4-*b′*]dipyran-4-one.

^1^H-NMR (400 MHz, chloroform-d, δ; ppm)

5.35 dd, 12.8, 3.2 Hz, H-2; 3.04 dd, 17.2, 12.8 Hz, H3α; 2.78 dd, 17.2, 3.2 Hz, H-3β; 12.03 s, 5 (-OH); 1.98 s, 6(-CH_3_), 7.39 d, 8.8 Hz, H-2′/H-6′; 6.95 d, 8.8 Hz, H-3′/H-5′; 3.84 s, 4′(-OCH_3_); 1.33 s, 2″(-CH_3_); 1.74 ddd, 6.8, 2 Hz, H-3″; 2.60 ddd, 6.8, 2 Hz, H-4″.

^13^C-NMR (100 MHz, chloroform-d, δ; ppm)

78.73 C-2; 43.55 C-3; 196.01 C-4; 100.34 C-4a; 159.82 C-5; 105.38 C-6; 7.18 6(-CH_3_); 161.02 C-7; 114.21 C-8; 158.66 C-8a; 131.20 C-1′; 127.59 C-2′/C-6′; 114.215 C-3′/C-5′; 158.66 C-4′; 55.59 4′(-OCH_3_); 76.13 C-2″; 26.84 2″(-CH_3_); 27.58 2″(-CH_3_); 32.04 C-3″; 16.77 C-4″.

IR; 3754.3 cm^−1^, 3436.2 cm^−1^, 2928.4 cm^−1^, 2375.8 cm^−1^, 1628.6 cm^−1^, 1516.6 cm^−1^, 1456.0 cm^−1^, 1347.4 cm^−1^, 1248.8 cm^−1^, 1166.6 cm^−1^, 1120.2 cm^−1^, 1033.2 cm^−1^, 892.0 cm^−1^, 827.5 cm^−1^, 573.7 cm^−1^.

HR-MS (ESI), calcd. for C_22_H_24_O_5_: [M^+^H^+^] 369.1623, found: 369.1696 ([M^+^H^+^]).

#### 4.7.4. Preparation of Vinylogous Cyclization Analogues (**1d**) and (**2d**)

Natural flavanones (**1**, 20 mg, 0.0591 mmol) and (**2**, 20 mg, 0.0543 mmol) were treated with DDQ (94 mg, 1:4 molar ratio) under reflux in 10 mL of sodium-dried benzene at 78 °C for 4 h. The crude reaction mixtures were submitted to TLC, eluted with a 3:7 n-hexane:CH_2_Cl_2_ system, to afford the vinylogous-cyclized analogues [22].

Vinylogous-cyclization analogue (**1d**):

(2*S*)-5-hydroxy-6,8,8-trimethyl-2-phenyl-2,3-dihydro-4*H*,8*H*-benzo [1,2-*b*:3,4-*b′*]dipyran-4-one.

^1^H-NMR (400 MHz, chloroform-d, δ; ppm)

5.38 dd, 12.8 3.2 Hz, H-2; 2.99 dd, 16.8, 12.8 Hz, H-3α; 2.80 dd, 16.8, 3.2 Hz, H-3β; 12.19 s 5(OH); 1.96 s, 6(-CH_3_); 7.42 m, -C6H5; 6.60 D, 10 Hz, H1″, 5.47 d, 10 Hz, H-2″;1.42 s, 3″ (-CH_3_);1.40 s, 3″ (-CH_3_).

^13^C-NMR (100 MHz, chloroform-d, δ; ppm)

78.80 C-2; 43.66 C-3; 196.21 C-4; 102.70 C-4a; 159.52 C-5; 102.80 C-6; 7.70 6(-CH_3_); 160.14 C-7; 104.72 C-8; 156.37 C-8a; 138.98 C-1′; 126.70 C-2′/C-6′; 128.91 C-3′/C-5′; 128.70 C-4′; 115.77 C-1″; 126.09 C-2″; 128.70 C-3″; 28.77 3″(-CH_3_); 28.67 3″(-CH_3_).

IR; 3433 cm^−1^, 2927 cm^−1^, 2871 cm^−1^, 2366 cm^−1^, 1735 cm^−1^, 1637 cm^−1^, 1458 cm^−1^, 1372 cm^−1^, 1292 cm^−1^, 1166 cm^−1^, 1124 cm^−1^, 981 cm^−1^, 901 cm^−1^, 762 cm^−1^, 695 cm^−1^, 480 cm^−1^.

HR-MS (ESI), calcd. for C_21_H_20_O_4_: [M^+^H^+^] 337.1361, found: 337.1439 ([M^+^H^+^]).
Vinylogous-cyclized analogue (**2d**):

(2*S*)-5-hydroxy-2-(4′-methoxyphenyl)-6,8,8-trimethyl-2-phenyl-2,3-dihydro-4*H*,8*H*-benzo [1,2-*b*:3,4-*b′*]dipyran-4-one.

^1^H-NMR (400 MHz, chloroform-d, δ; ppm)

5.35 dd, 12.8, 3.2 Hz, H-2; 3.05 dd, 17.2, 12.8 Hz, H-3α; 2.79 dd, 17.2, 3.2 Hz, H-3β; 12.39 s, 5 (-OH), 1.98 s, 6(-CH_3_), 7.38 d, 8.4 Hz, H-2′/H-6′; 6.95 d, 8.8 Hz, H-3′/H-5′; 3.84 s, 4′(-OCH_3_); 1.459 s, 2″(-CH_3_); 5.46 d, 10 Hz, H-3″, 6.55 d, 10 Hz, H-4″.

^13^C-NMR (100 MHz, chloroform-d, δ; ppm)

78.92 C-2; 43.52 C-3; 196.10 C-4; 102.71 C-4a; 159.90 C-5; 104.64 C-6; 7.69 6(-CH_3_); 161.37 C-7; 105.82 C-8; 159.83 C-8a; 130.99 C-1′; 126.20 C-2′/C-6′; 114.22 C-3′/C-5′; 106.07 C-4′; 55.56 4′(OCH_3_); 78.24 C-2″; 28.82 2″(CH_3_); 28.63 2″(CH_3_); 127.68 C-3″; 116.10 C-4″.

IR; 3437.8 cm^−1^, 2975.0 cm^−1^, 2925.7 cm^−1^, 2375.5 cm^−1^, 1634.5 cm^−1^, 1515.8 cm^−1^, 1456.0 cm^−1^, 1373.1 cm^−1^, 1299.5 cm^−1^, 1173.0 cm^−1^, 1124.3 cm^−1^, 1031.5 cm^−1^, 901.9 cm^−1^, 829.6 cm^−1^, 692.3 cm^−1^, 500.3 cm^−1^.

HR-MS (ESI), calcd. for C_22_H_22_O_5_: [M^+^H^+^] 367.1467, found: 367.1443 ([M^+^H^+^]).

### 4.8. Data Analysis

In vivo experiments were conducted in triplicate, and data are expressed as mean ± standard deviation (SD). Group differences were evaluated using one-way ANOVA followed by Tukey’s post hoc test.

## 5. Conclusions

Eight analogues (**1a**–**1d**) and (**2a**–**2d**) were prepared and characterized from natural flavanones (**1**) and (**2**) through a pharmacomodulation strategy. Six of these compounds (**1a**), (**1b**), (**2a**), (**2b**), (**2c**), and (**2d**) are novel structures. Among them, analogues (**1c**), (**2c**), and (**2d**) demonstrated significant anti-inflammatory activity in an in vivo model of mouse ear edema induced by TPA, with analogue (**2c**), (2*S*)-5-hydroxy-2-(4′-methoxyphenyl)-6,8,8-trimethyl-2-phenyl-2,3,9,10-tetrahydro-4*H*,8H-benzo [1,2-*b*:3,4-*b’*]dipyran-4-one, showing the highest inhibition (98.62 ± 1.92%).

These results highlight the relevance of structural modifications such as cyclization, methoxylation, and prenylation in enhancing lipophilicity, membrane permeability, and overall pharmacological efficacy, while remaining consistent with drug-likeness criteria (Lipinski’s Rule of Five). The correlation observed between lipophilicity (Log P) and biological activity underscores the importance of fine-tuning physicochemical properties to optimize pharmacokinetics without compromising safety.

From a medicinal chemistry perspective, the study demonstrates that semi-synthetic derivatization of natural flavanones is a valuable approach to generate more potent and selective anti-inflammatory agents. Among these, analogue (**2c**) emerges as a promising lead compound, warranting further investigation into its mechanism of action, selectivity, and safety profile.

Overall, this work provides both experimental validation and rational design principles that may guide the future development of flavanone-based scaffolds as therapeutic candidates for inflammatory disorders.

## Figures and Tables

**Table 1:** Physicochemical properties for the natural flavanones (**1**) and (**2**), their analogues (**1a**–**1d**) and (**2a**–**2d**) and indomethacin.

Physicochemical Properties	Compounds										
	(1)	(1a)	(1b)	(1c)	(1d)	(2)	(2a)	(2b)	(2c)	(2d)	Indomethacin
Total Energy (kcal/mol)	−98,539.800	−126,384.000	−102,117.000	−98,543.600	−97,886.078	−109,514.032	−137,357.701	−113,091.124	−109,517.615	−108,860.185	−104,099.550
Bond Energy (kcal/mol)	−5090.910	−6159.707	−5356.437	−5094.670	−4962.790	−5463.810	−6532.550	−5729.163	−5467.390	−5335.570	−4548.765
Heat of Formation (H°f) (kcal/mol)	−117.740	−175.450	−108.173	−121.500	−93.820	−155.990	−213.640	−146.246	−159.670	−131.950	−87.997
Dipole Moment (µ)	4.263	1.981	2.079	4.262	3.770	4.562	2.649	3.525	4.930	4.220	1.996
HOMO (eV)	−8.860	−9.030	−8.830	−8.760	−8.610	−8.854	−9.033	−8.837	−8.751	−8.600	−8.755
LUMO (eV)	−0.482	−0.660	−0.644	−0.4	−0.479	−0.469	−0.653	−0.633	−0.389	−0.464	−0.857
Molecular Surface Area (A^2^)	567.310	681.540	587.830	564.470	561.650	608.150	721.54	641.400	607.700	606.060	504.830
Molecular Weight (MW) (uma)	338.400	428.480	352.430	338.400	336.400	368.430	452.500	382.460	368.430	366.410	357.790
Molecular Volume (MV) (A^3^)	979.300	1199.640	1029.93	968.9	961.130	1054.850	1275.400	1121.140	1045.830	1083.010	960.940
Logarithm of the Partition Coefficient (Log P)	0.850	0.420	0.880	0.200	0.090	−0.140	−0.580	−0.110	−0.790	−0.910	−1.430

**Table 2:** In silico predicted ADME properties of natural flavanones (**1**) and (**2**) their analogues (**1a**–**1d**) and (**2a**–**2d**) and Indomethacin.

Compounds	Pharmacokinetics					
	GI Absortion	BBB Permeation	CYP450 Inhibition	TPSA (Å^2^)	Log P	Water Solubility (Log S)
(**1**)	High	Yes	CYP1A2: Yes CYP2C19: Yes CYP2C9: Yes CYP2D6: No CYP3A4: Yes	66.76	3.97	−5.27
(**1a**)	High	No	CYP1A2: No CYP2C19: Yes CYP2C9: Yes CYP2D6: No CYP3A4: Yes	78.90	4.46	−5.24
(**1b**)	High	Yes	CYP1A2: No CYP2C19: Yes CYP2C9: Yes CYP2D6: No CYP3A4: Yes	55.75	4.34	−5.48
(**1c**)	High	Yes	CYP1A2: Yes CYP2C19: Yes CYP2C9: Yes CYP2D6: Yes CYP3A4: Yes	55.76	3.91	−5.03
(**1d**)	High	Yes	CYP1A2: Yes CYP2C19: Yes CYP2C9: No CYP2D6: No CYP3A4: Yes	55.76	2.97	−3.79

**Table 3:** In silico predicted ADME properties of natural flavanones (**1**) and (**2**) and their analogues (**1a**–**1d**) and (**2a**–**2d**).

Compounds	Pharmacokinetics					
	GI Absortion	BBB Permeation	CYP450 Inhibition	TPSA (Å^2^)	Log P	Water Solubility (Log S)
(**2**)	High	No	CYP1A2: No CYP2C19: Yes CYP2C9: Yes CYP2D6: No CYP3A4: Yes	75.99	3.99	−5.35
(**2a**)	High	No	CYP1A2: No CYP2C19: Yes CYP2C9: Yes CYP2D6: No CYP3A4: Yes	88.13	4.45	−5.33
(**2b**)	High	Yes	CYP1A2: No CYP2C19: Yes CYP2C9: Yes CYP2D6: No CYP3A4: Yes	64.99	4.36	−5.56
(**2c**)	High	Yes	CYP1A2: No CYP2C19: Yes CYP2C9: Yes CYP2D6: Yes CYP3A4: Yes	64.99	3.88	−5.11
(**2d**)	High	Yes	CYP1A2: No CYP2C19: No CYP2C9: No CYP2D6: No CYP3A4: Yes	64.99	2.67	−3.88
**Indomethacin**	High	No	CYP1A2: Yes CYP2C19: No CYP2C9: Yes CYP2D6: No CYP3A4: No	77.76	3.35	−4.66

**Table 4:** Biological activities predicted by PASS Online for natural flavanones (**1**) and (**2**) and their analogues (**1a**–**1d**) and (**2a**–**2d**) related to inflammation.

Compounds	Anti-Inflammatory	NOS2 Expression Inhibitor	Free Radical Scavenger	Lipid Peroxidase Inhibitor	Antioxidant	HMOX1 Expression Enhancer	MMP9 Expression Inhibitor	Histamine Release Inhibitor	Cytoprotectant
(**1**)	0.782	0.860	0.857	0.922	0.778	0.693	0.672	0.672	0.627
(**1a**)	0.803	0.686	0.793	0.925	0.678	0.631	ND	0.617	0.659
(**1b**)	0.740	0.848	0.896	0.927	0.697	0.665	0.661	0.639	0.632
(**1c**)	0.743	0.715	0.786	0.930	0.780	0.617	ND	0.556	0.588
(**1d**)	0.630	0.861	0.844	0.790	0.759	0.620	0.449	0.568	0.612
(**2**)	0.754	0.899	0.917	0.937	0.716	0.647	0.733	0.635	0.634
(**2a**)	0.776	0.772	0.868	0.938	0.633	0.567	0.512	0.585	0.665
(**2b**)	0.734	0.868	0.914	0.935	0.692	0.647	0.678	0.631	0.634
(**2c**)	0.711	0.806	0.858	0.943	0.717	0.551	0.502	0.531	0.598
(**2d**)	ND	0.746	0.633	ND	0.547	0.516	ND	ND	0.605
**Indomethacin**	0.711	0.440	0.151	0.410	ND	0.298	0.231	0.242	0.285

ND: not detectable; MMP9: matrix metalloproteinase-9.

**Table 5:** Inflammation inhibition percentage of natural flavanones (**1**) and (**2**) and analogues (**1a**–**1d**) and (**2a**–**2d**).

Compounds	Inflammation Inhibition Percentage (%)
(**1**)	12.20 ± 1.12 ^g^
(**1a**)	16.02 ± 1.47 ^g^
(**1b**)	36.66 ± 2.02 ^ef^
(**1c**)	71.64 ± 1.86 ^c^
(**1d**)	37.79 ± 1.83 ^ef^
(**2**)	68.35 ± 1.45 ^d^
(**2a**)	41.75 ± 2.07 ^e^
(**2b**)	25.69 ± 1.59 ^f^
(**2c**)	98.62 ± 1.92 ^a^
(**2d**)	76.12 ± 1.74 ^c^
**Indomethacine**	91. 00 ± 0.46 ^b^

Inflammation inhibition percentages (%) are expressed as mean ± SD (*n* = 3). Different superscript letters (a, b, c, d, e, f, g, ef) indicate statistically significant differences among groups (*p* < 0.05), as determined by one-way ANOVA followed by Tukey’s post hoc test. Values sharing the same letter or combination of letters are not significantly different from each other.

**Table 6:** Summary of SAR features contributing to anti/inflammatory activity of flavanones analogues.

Structural Feature	Effect on Activity	Supporting Evidence
Cyclization/rigidity	↑ target binding, ↓ entropic penalty	(**2c**) > (**1**), (**2**) (Section 3.5)
Methoxy group at 4′	↑ lipophilicity, permeability, stability	(**2c**) > (**1c**) (Section 3.4)
Conjugated double bond (**2d**)	↓ activity, ↑ reactivity, potential metabolite formation	(**2d**) < (**2c**) (Section 3.5)

Note: ↑ indicates an increase, and ↓ indicates a decrease in the respective property (e.g., ↑ target binding, ↓ entropic penalty).

## Data Availability

The data presented in this study are available on request from the corresponding author.

## Supplementary Materials

The following supporting information can be downloaded at: https://www.mdpi.com/article/10.3390/molecules30183728/s1.
