# Supplementary material for: In Vivo Anti-Inflammatory Evaluation and In Silico Physicochemical Characterization of Flavanones from E. platycarpa Leaves
Source: Molecules. 2025 Sep 13;30(18):3728. doi: 10.3390/molecules30183728 (PMC12472972; doi:10.3390/molecules30183728)
Supplement: Supplementary file 1 [file molecules-30-03728-s001.zip › molecules-3842225-supplementary.pdf]

molecules

MDPI

Supporting Information

In Vivo Anti-Inflammatory Evaluation and In Silico Physicochemical Characterization of Flavanones from *E. platycarpa* Leaves

Berenice Andrade-Carrera[1,*,†], Valeri Domínguez-Villegas[2,*,†], Ana Calpena[3] and María Luisa Garduño-Ramírez[4]

1 Facultad de Nutrición, Universidad Autónoma del Estado de Morelos, Cuernavaca 62350, Morelos, Mexico
2 Facultad de Ciencias Químicas e Ingeniería, Universidad Autónoma del Estado de Morelos, Cuernavaca 62210, Morelos, Mexico
3 Department of Pharmacy, Pharmaceutical Technology and Physical Chemistry, School of Pharmacy and Food Sciences, University of Barcelona, 27-31 Joan XXIII Av., 08028 Barcelona, Spain
4 Centro de Investigaciones Químicas, Instituto de Investigación en Ciencias Básicas y Aplicadas, Universidad Autónoma del Estado de Morelos, 62210 Cuernavaca, Morelos, Mexico; lgarduno@uaem.mx
* Correspondence: bereniceac@uaem.mx (B.A.-C.); valeri.dominguez@uaem.mx (V.D.-V.)
† These authors contributed equally to this work.

S3. Plausible Mechanisms

S3.1. Acetylation

The acetylation of natural flavanones (**1**) and (**2**) was performed using acetic anhydride and pyridine, affording analogues (**1a**) and (**2a**). A plausible reaction mechanism is illustrated in Scheme S1.

 https://doi.org/10.3390/molecules30183728

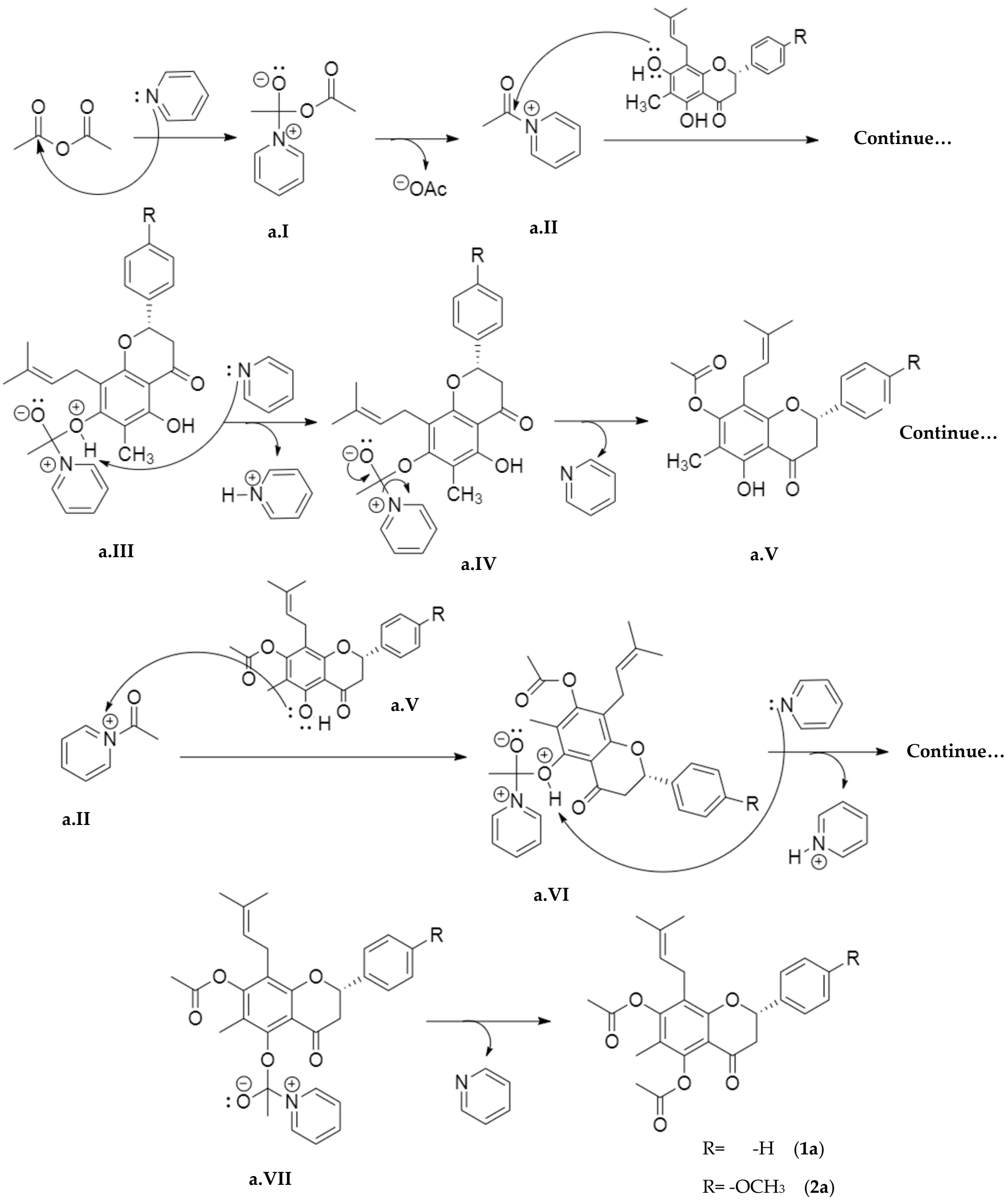

Scheme S1. A plausible mechanism for the semi-synthesis of acetylated analogues (**1a**) and (**2a**).

S3.2. Methylation

Methylation of flavanones (**1**) and (**2**) was carried out using diazomethane, yielding analogues (**1b**) and (**2b**). The proposed mechanism involves nucleophilic attack of the hydroxyl group on diazomethane and subsequent formation of the methyl ether (Scheme S2).

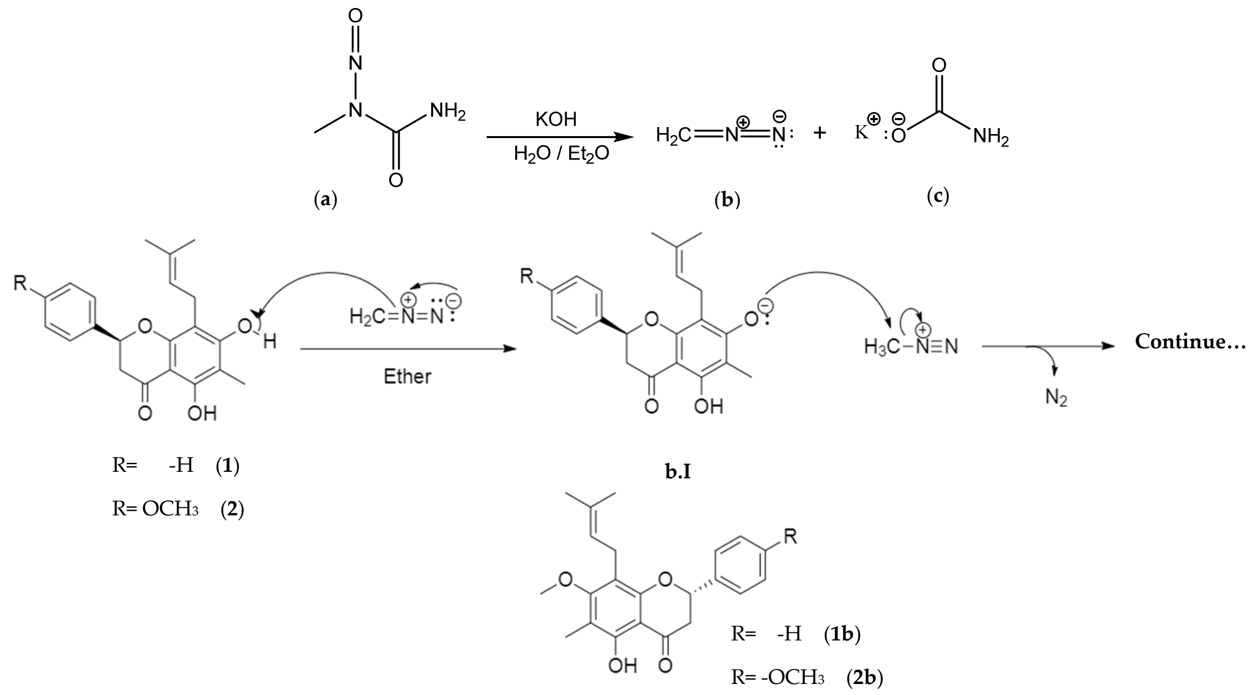

Scheme S2. A proposed mechanism for semi-synthesis of methylated analogues (**1b**) and **2b**).

S3.3. Cyclization

Cyclization of prenylated flavanones (**1**) and (**2**) leads to analogues (**1c**) and (**2c**). The mechanism involves carbocation formation followed by intramolecular nucleophilic attack of the hydroxyl oxygen (Scheme S3).

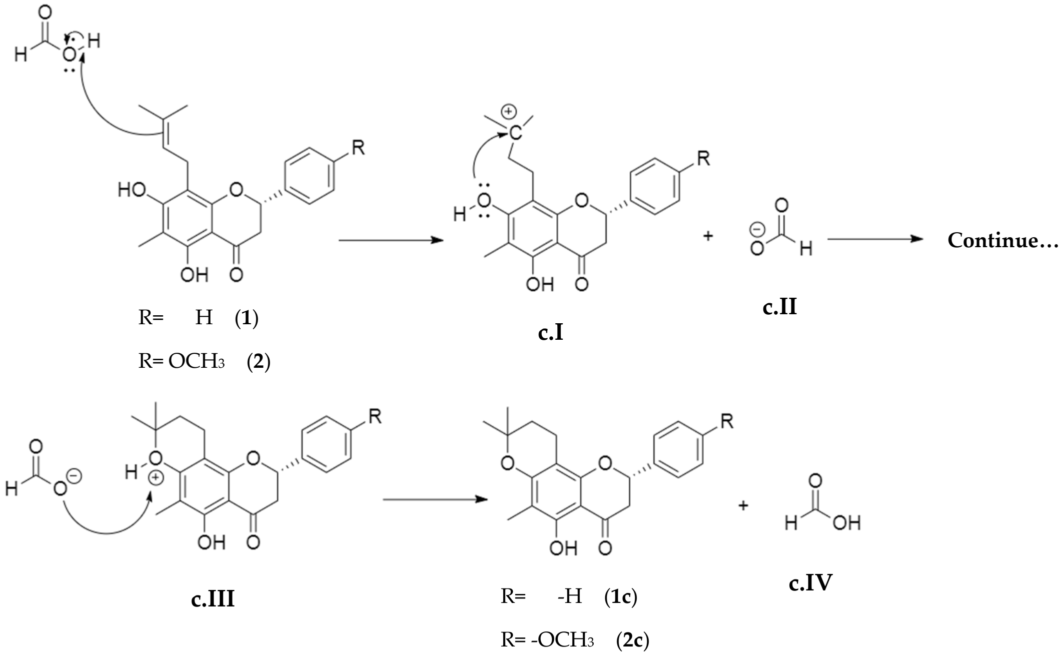

Scheme S3. A plausible mechanism for semi-synthesis of cyclized analogues (**1c**) and (**2c**).

S3.4. Vinylogous-cyclization

Vinylogous cyclization of flavanones (**1**) and (**2**) using DDQ under anhydrous conditions affords analogues (**1d**) and (**2d**). The mechanism involves dehydrogenation and cyclization as illustrated in Scheme S4.

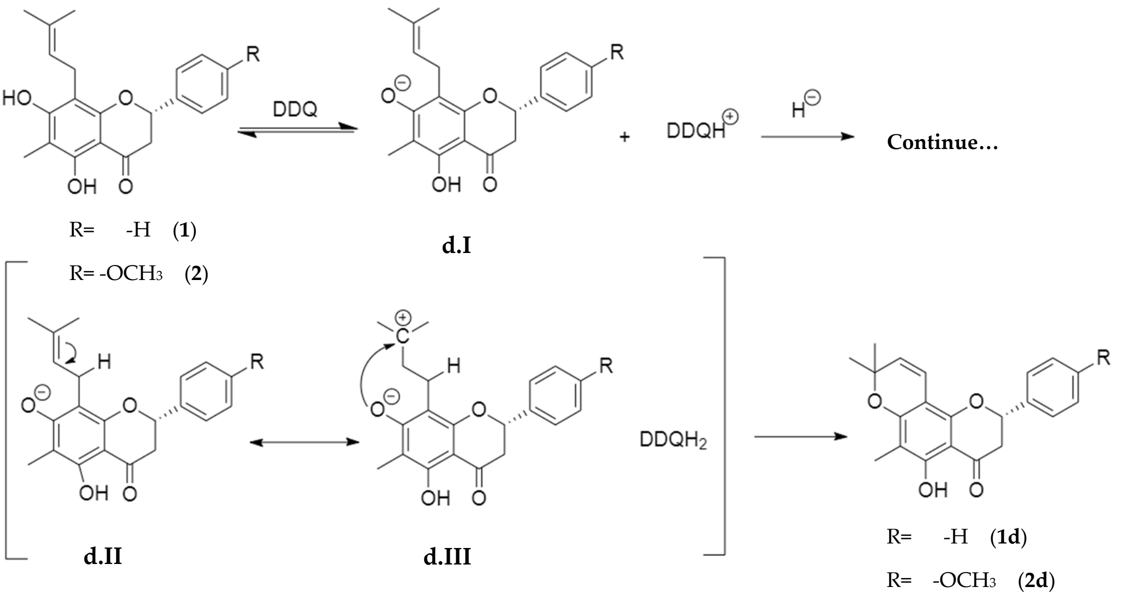

Scheme S4. A proposed mechanism for semi-synthesis of vinylogous-cyclized analogues (**1d**) and (**2d**).
